# Cluster Analysis Classification of Honey from Two Different Climatic Zones Based on Selected Physicochemical and of Microbiological Parameters

**DOI:** 10.3390/molecules26082361

**Published:** 2021-04-19

**Authors:** Elżbieta Rosiak, Beata Madras-Majewska, Dariusz Teper, Anna Łepecka, Dorota Zielińska

**Affiliations:** 1 Department of Food Hygiene and Quality Management, Institute of Human Nutrition, Warsaw University of Life Sciences in Warsaw—SGGW, Nowoursynowska str. 166, 02-787 Warsaw, Poland; anna_lepecka@sggw.edu.pl (A.Ł.); dorota_zielinska@sggw.edu.pl (D.Z.); 2 Apiculture Division, Institute of Animal Sciences, Warsaw University of Life Sciences in Warsaw—SGGW, 02-787 Warsaw, Poland; beata_madras_majewska@sggw.edu.pl; 3 Apiculture Division in Puławy, The Research Institute of Horticulture, 24-100 Puławy, Poland; dariusz.teper@inhort.pl

**Keywords:** honey, microbiological quality, probiotic, geographical origin, water content

## Abstract

The geographical origin of honey affects its composition, which is of key importance for the health-promoting properties and safety of the product. European regulations clearly define the physicochemical requirements for honey that determine the microbiological quality. On the other hand, legislation abolishes microbiological criteria. In the study 40 honey samples originating from two different climatic zones were analyzed. The water content, pH, water activity analysis and the microbiological quality of honey samples have been tested using the reference plate method (total viable count, yeast and molds, lactic acid bacteria, *Bacillus* spp.). The cluster classification showed that total viable count of bacteria could be used as a measure alternative to the count of *Bacillus* spp. and 70% of honeys from the tropical climate zone had different microbiological quality than honeys from the temperate climate zone but still under the level 3.0 log cfu/g. The study has revealed that geographical origin of honey may significantly affect the quality and safety of honey. It was considered that water content can be the most informative and handy marker of the microbiological quality of honeys. Analysis of lactic acid bacteria showed temperate climate zone honeys as a source of beneficial bacteria in the diet.

## 1. Introduction

The properties of honey result from their antimicrobial, antioxidant, enzymatic dietary and sensory properties, as well as prebiotic effects and the presence of probiotic bacteria [1,2]. Some strains of lactic acid bacteria have a beneficial effect on the human body by normalizing the microbiota of the gastrointestinal tract and the body’s antiallergic response [3,4]. The analysis of the presence of the 16S rRNA gene of *Lactobacillus* bacteria confirmed the presence of these bacteria in a large percentage of bee products, respectively: in honey—90.9%; in pollen—70.6%; in propolis—83.9%. *Lactobacillus kunkeei* was the dominant species (98%) of *Lactobacillus* spp. in bee products [5]. It was also proved that the *Lactobacillus kunkeei* YB38 strain promoted the production of IgA antibodies in humans. In vitro studies showed that strains YB83 and YB38 present in bee pollen increased the production of IgA antibodies in Peyer cells in mice and showed mitogenic activity. Depending on the species and strain of *Lactobacillus*, the immunomodulatory effects may vary, and these strains can safely improve the immune responses of the human body [5]. It has been shown that lactic acid bacteria have a beneficial effect on health with a concentration of at least 10^9^ cfu/g. However, confirmation of the probiotic effect requires detailed research [6].

Among the major components of honey, the next can be reported: sugars, water, ni-trogenous substances, proteins, organic acids and polyphenols. Honey sugars are a complex mixture of carbohydrates dominated by fructose and glucose [7,8,9,10,11,12]. 

The composition of honey and the content of compounds influencing the properties of honey depends on many factors: the botanical origin of honey, environmental and climatic factors, as well as the honey extraction process [11,13,14].

The microbiota of honey also results on its chemical composition depending on the botanical, environmental and climatic factors. Beekeepers from the tropics, where the vegetation period is long, can harvest large quantities of honey almost all year round. However, high ambient temperature and humidity levels impede the aging, while the environment promotes the growth of bacteria and yeasts. Typically, excess water is removed from honey through heating, which leads to thermal stress, consequently, deterioration of health-promoting properties and succulence. On the other hand, in the moderate climate, the harvesting is conducted a few times, at sufficient intervals, over an approximately half-year period which favors the natural ripening process [9]. An element of the health quality of honey is microbiological safety. Ripe honeys are dominated by aerobic bacteria, while yeast and molds are in the minority. Identification of microbiome of unripe honey showed the presence of Gram-positive bacteria mainly *Lactobacillus* (1.0 × 10^2^–1.2 × 10^3^/g) and genera: *Bacillus, Staphylococcus* and *Enterococcus*. Gram-negative are represented by *Gluconobacter* bacteria (6.0 × 10^2^–7.0 × 10^3^/g), and intrinsic biota of bees which in 75% consists of Enterobacteriaceae (*E. coli, Salmonella, Shigella, Klebsiella, Proteus, Serratia, Citrobacter* and *Edwardsiella, Erwinia*) [15,16]. Secondary sources of microbes in honey, are likely to be the same as for other foods (humans, animals, water, soil, air and/or processing facilities or equipment, such as honey harvesting or storage containers) and, consequently, appropriate standards of hygiene must be applied in all operations involving honey handling [17]. *Clostridium* spp., *Corynebacterium* spp., *Bacillus* spp. and *Pseudomonas* spp. are bacteria commonly found in soil. *Brochotrix* spp., *Citrobacter* spp., *Enterobacter* spp., *Lactobacillus* spp., *Lactococcus* spp., *Pediococcus* spp., *Listeria* spp. and *Flavobacterium* are found in plants and plant products. Air and dust are important source of *Bacillus* spp., *Clostridium* spp. and *Micrococcus* spp. species. *Saccharomycses* and *Torula* yeasts can be found in high-moisture sugars [15,16]. The ripe honeys contain mostly molds and different species of sporulating bacteria *Clostridium* and *Bacillus* (because of their possibility of development in conditions of limited water availability; a_w_ 0.57–0.62) and osmophilic yeast (*Schizosaccharomyces, Hansenula, Torula, Pichia, Nemaiospora, Schwanniomyces* and *Rhodotorula*). The nectar honey types are dominated by the *Saccharomyces* yeasts, represented by a dozen or so strains and aerobic and anaerobic bacterial spores [15]. On the other hand, another safety issue identified in honey are chemical pollution, the source of which are improper practices in agribusiness. Many authors indicated that the presence of pesticides in honeys can be serious problem [8].

In European countries there is a lack of the national microbiological guidelines for assessing the microbiological quality of honey and bee products, abolished by European Union regulations. The microbiological quality of honey is assessed according to the criteria adopted for food and feed in accordance with Regulation (EC) No 178/2002 of the European Parliament and of the Council [18] applicable to all stages of production, processing and distribution of food and feed as well as in Food Code (Codex Alimentarius) where the microbiological quality of honey relates to the criteria established in accordance with the principles and guidelines for the determination and application of microbiological and hygiene criteria related to food [19]. The quality of the honey is defined by physicochemical criteria well compiled by European Directive 2014/63/EU, while the microbiological aspects are ignored [20]. Under this Directive also there is no requirement for disclosing the country of origin on a honey package [21]. Where the product is a blend originating from more than one EU and/or non-EU country, it may be designated as an “blend of EU honey”, “blend of non-EU honey” or “blend of EU and non-EU honey” [22]. Because of the heavy reliance of the product quality on its origin, the consumer should not be “geographically misguided”. The modern consumer has the possibility to choose products from a very wide range and markets in many retail countries, and the role of information provided with the product is growing. When making a choice, the consumer can take into account the origin of the product, its safety and health quality [23].

The aim of the research was to evaluate and classify honey samples from different climatic zones on the basis physicochemical properties and the evaluation of their microbiological quality and safety. Most of the research publications concerns physicochemical properties of honey in the light of the EU Directive [21]. Honey microbiota research is mainly focused on the analysis of the occurrence of toxynogenic *Clostridium botulinum* bacteria [12,24,25,26]. Only the small number of current scientific publications on the microbiological and hygienic quality of honeys are available. To our best knowledge these are the first honey study that present a microbiological assessment in comparison to the physicochemical requirements.

## 2. Results

### 2.1. Pollen Analysis

The analyzed honey samples had been declared by the beekeepers as varietals honeys, based on organoleptic features as well as bee forage (available to bees flowering surrounding plants) (Table 1). However, the pollen samples analyze showed that (in all samples predominant pollen > 45% was not identified), so all the samples were considered as multifloral honeys [27,28,29]. 

### 2.2. Physicochemical Analysis

The physicochemical results of honey samples analysis were presented in detail in Table 2. The pH value of Polish and Thai honey samples ranged from 3.38 to 4.43 and 3.44 to 4.90, respectively. The Polish honey samples were characterized by very balanced water activity. The water activity of Polish honey samples ranges from 0.501 to 0.578. The median value of a_w_ (0.535) differed from the average value (0.534) by 0.001 which is confirmed by the even distribution of this parameter in the tested samples. Thai honeys samples a_w_ values were found significantly higher (*p* < 0,05) than Polish one; the results ranged from 0.553 to 0.673, the average and median value of aw were 0.605 and 0.609, respectively. Moreover, most of the Polish honey samples contained up to 20% of water. This limit was exceeded in samples P1, P8 and P16 (slightly, by 0.5–0.9%) and in sample P3 (by 2%). For the Thai honeys, thirteen of the twenty samples were above the limit (by more than 6% for samples T12 and T20). 

### 2.3. Microbiological Analysis

**Figure 1:**
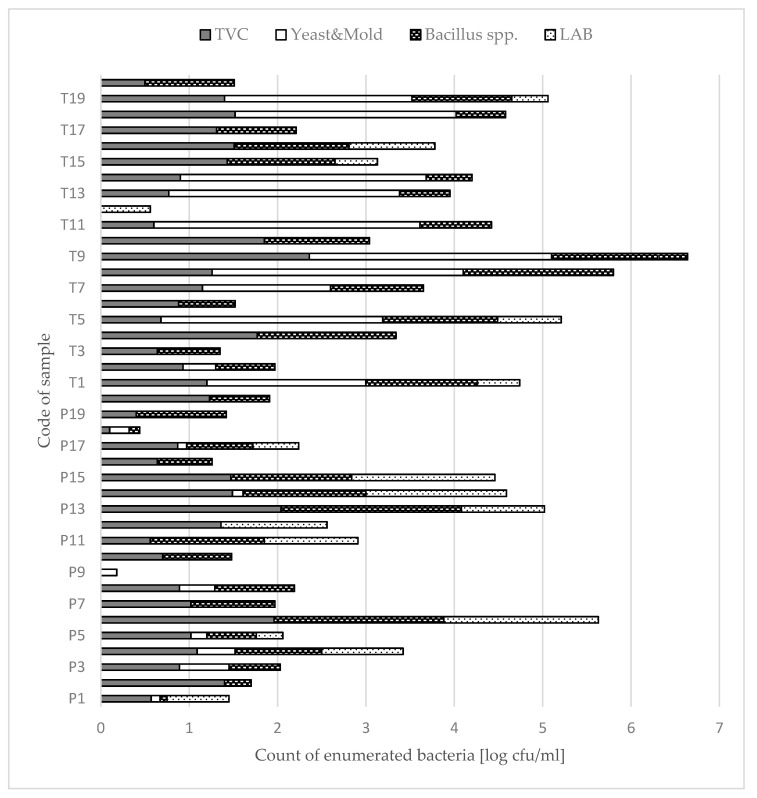
Results of microbiological analyses of Polish and Thai honeys.

The results of microbiological analysis were shown at Figure 1. The bacteria of *Salmonella* spp. was not detected in any of the tested honey samples. The mean values obtained for the total number of mesophilic aerobic bacteria (TVC) in the examined honey samples were 0.98 log cfu/g and 1.13 log cfu/g for Polish and Thai honeys, respectively. The yeasts and molds were found in 11 of Thai and 9 of Polish honeys samples, the mean count of them was respectively 1.24 and 0.11 log cfu/g. Thai honey samples (30%) contained a small amount of lactic acid bacteria (LAB), not exceed 0.97 log cfu/g. The Polish honey samples (50%) contains the population of LAB ranged from 0.30 log cfu/g up to 1.75 log cfu/g. In the case of mesophilic spores of the *Bacillus* spp. bacterium, the mean value obtained in the Polish honeys samples was 0.82 log cfu/g, and for Thai honey samples the mean value was 0.98 log cfu/g.

**Figure 2:**
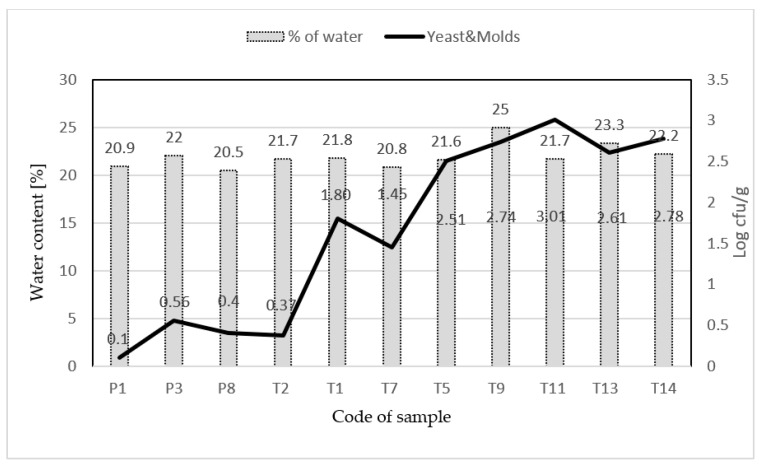
Water content [%] and the number of yeasts and molds [log cfu/g] in Polish (P) and Thai (T) honeys, in which yeast and molds were detected.

Figure 2 shows the relationship between water content and the number of yeasts and molds only in samples where yeasts and molds have been detected. In the case of three samples of Polish honeys, the excessive water content did not affect the number of yeasts and molds, which not exceeding 0.5 log cfu/g, while in six samples of Thai honeys, the increased water content caused high number of yeast and molds 1.8–3.01 log cfu/g. The correlation between the number of yeast and molds and water content was 0.575 and 0.428 for Polish and Thai honey samples respectively, it has also clearly seen that in some cases water content can affect yeast and molds dynamic growth.

**Figure 3:**
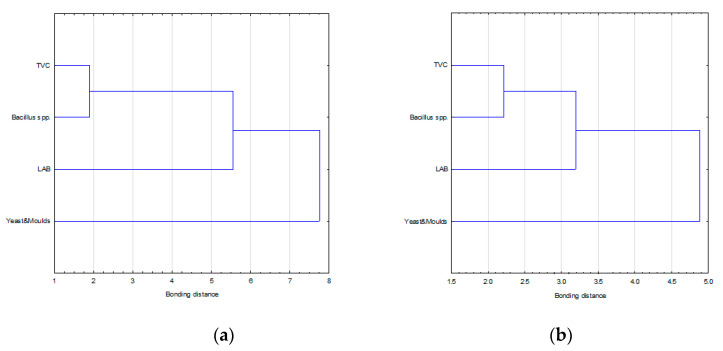
Dendrogram of hierarchical cluster analysis of microbiological variables determined by the Ward method in Thai honeys (**a**) and Polish honeys (**b**).

**Figure 4:**
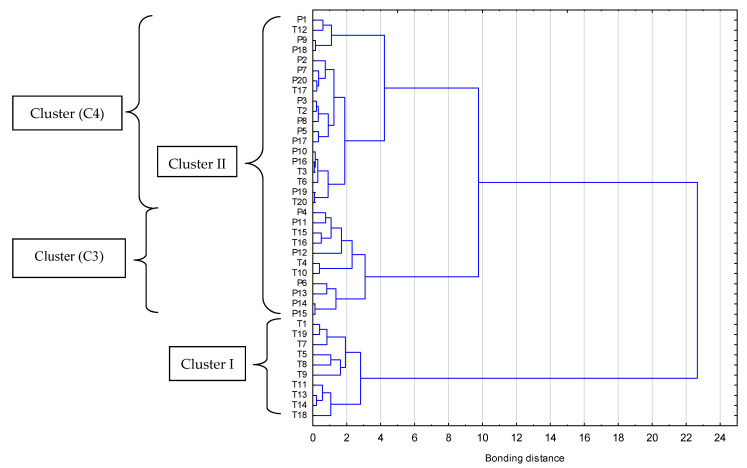
Dendrogram of hierarchical cluster analysis of Polish and Thai honeys cases determined by the Ward method.

Figure 3 and Figure 4 shows the results of cluster analysis classification (variable and cases, respectively) of the Polish and Thai honeys based on results of four microbiological analysis (TVC, count of yeast and molds, LAB, *Bacillus* spp.).

Based on an analysis of the classification of variables for the Polish and Thai honeys, the counts of mesophilic aerobic microorganisms and the *Bacillus* spp. form mutually correlated pairs and the results can be used interchangeably for microbiological quality evaluation of the honeys irrespective of the cluster merging method. The variable—LAB represented a distinct cluster, slightly correlated to the variables: TVC and the *Bacillus* spp., particularly for the Thai honeys. The variable—yeasts and molds represented a different cluster (Figure 3).

Figure 4 shows the classification of cases of the Polish and Thai honeys. The analysis identified two main groups (Cluster I and Cluster II) of honeys with a distance of 20 between cluster centers, which attests to a significant variation of the features of the honeys. Ten Thai honey samples, varying significantly in their microbiological contamination levels, were classified as belonging to the first major cluster (CI). Similar to each other in this group were the honeys: T13 and T14 as well as T1 and T19. The second major cluster (CII) was formed from two smaller clusters marked as C3 and C4, with a binding length of approximately nine. The C3 groups of honey with similar microbial contamination as in group (CI) included 11 honey samples: 4 Thai and 7 Polish. The most similar in terms of microbiological quality in this group were multiflorous Polish honeys P14 and P15, Thai honeys constituted 36.4% in this group (T4, T10, T15, T16). The last, third, cluster (C3) consisting 19 samples featured the smallest variation in their microbiological quality including contamination. Thai honeys (T2, T3, T6, T12, T17, T20) represented 31.5% of the samples from this cluster. The classification of honey originating from the two climatic zones demonstrated significant differences −70% of the tropical honeys (C1 and 4 samples of C3) had different microbiological quality than honey from temperate climate zone.

## 3. Discussion

Pollen analysis of honey is widely used to verify the claimed geographic and floral origin of honey samples [30]. Beekeepers do not always perform pollen analysis due to cost, lack of the necessary equipment and knowledge. Based on the literature data, it is known that beekeepers declare the type of honey on the basis of the organoleptic characteristics and properties of the bee forage [31,32]. On the other hand, honey with the presence of one type of pollen (predominant pollen) at the level above 45% [27,28,29] can be considered as varietal honey. In case of the present study, future, more detailed analyzed should be performed to determinate the predominant pollen and type of tested honey samples.

The data obtained from physicochemical study indicated that honey samples originating from the two different climatic zones (transitional between the continental moderate and oceanic moderate one vs. monsoon tropical) differ in case of pH, water activity and water content. In the present study, the pH value of the honeys of Polish and Thai samples (Table 2) were 3.3–4.9, which is consistent with other authors findings [15,16,33]. None of the normative acts imposes a minimum or maximum pH or water activity values. The pH value depends on the acids present in the honey (e.g., acetic, butyric, citric, formic, gluconic, lactic and malic). It was found, that the gluconic acid present in the honey in the range of 0.23–0.98% plays the greatest role in antimicrobial activity of honey [7]. 

The water content decreases as the water evaporates during the honey maturation period. In the tropical climate zone, air humidity is high, which interferes with the natural ripening process. Due to the high sugar content (especially fructose and glucose) of honey, the osmotic pressure of honey is usually high leading to low water activity (a_w_) in reported range 0.56–0.62. The limiting water activity for growth of osmotolerant yeast is about 0.61–0.62 and much other microorganisms. Knowledge of water activity of honey is also needed to predict moisture exchange with the environment, since water activity is the driving force behind water transfer from/to honey [16,34]. Water activity (a_w_) and water content (%) of Polish honey samples was lower than Thai honey samples. The average and median value of Polish samples were identical, 0.535 and 0.534, respectively; in Thai samples increased value of a_w_ (average 0.605 and median 0.609 could lead to an increase in yeasts and molds count.

Moreover, most of the Polish honey samples contained up to 20% of water, which is in line with EU Directive [21]. This limit was exceeded in four Polish samples (P1, P3, P8 and P16) and in the thirteen samples of Thai honeys, which indicates the unripe of Thai honeys [17,21,27]. 

If the water content is high, the presence of a single mold cell may be sufficient to initiate a fermentation, causing adverse sensory changes. When yeasts and molds grow in honey, they break down monosaccharides which indicates a fermentation. As a result of this process, ethyl alcohol and carbon dioxide are formed. This is followed by a honey acidification due to the growth of bacteria, which cause the formation of acetic acid and non-volatile acids that have a characteristic aroma [35,36,37]. In the research of Madras–Majewska et al., 2016 one of three Thailand multifloral honey samples was negatively evaluated with respect to sensory attributes because of the tart, burning and sharp taste. The changes were connected with above 20% of water content (21.7%) and high count of yeasts and molds (more than 3.5 log cfu/g) [9]. 

In the present study the slight correlation between the number of yeast and molds and water content also was found. According to Snowdon and Cliver, 1996, yeast and molds activity is limited by the content of free water, causing that honey from humid regions is more prone to be contaminated with osmophilic yeasts [15]. Piana et al., 1991 reported yeast growth only in honey samples with a water activity < 0.65 [38]. The presence of yeasts and molds is also often caused by cross-contamination from product handling equipment [17].

Currently, the situation with the honey legislation complicates even more if we consider that some countries issues are national provisions, decisions and guidelines filling the gap in European and International legislation, despite the EU recommendations. Most of them set limits to define the physicochemical, organoleptic, microbiological and microscopic characteristics of monofloral honey, some have provisions regarding the country where the honey has been harvested and several others are differentiated from the set criteria. The differences among the national provisions enhance the difficulties of the applicability of honey regulations and make the necessity of uniformity of honey legislation [22]. In the repealed by EU low Polish legislation established in relation to bee products which are not honeys (pollen, propolis, bee bread), the microbiological criteria for the total number of mesophilic aerobic microorganisms were set at maximum level 5 × 10^4^ (4.7 log cfu/g); not more than 5 × 10^2^ (2.7 log cfu/g) of yeast and molds; not more than 100 (2 log cfu/g) of *Staphylococcus aureus* and aerobe *Bacillus cereus* [39]. Mexico, the major world honey exporter countries use no mandatory standard (Mexico NMX-036-Normex-2006) establishing the accepted presence of no more than 1000 cfu/g (3.0 log cfu/g) of non-pathogenic bacteria and up to 100 cfu/g (2.0 log cfu/g) of yeast and molds. Similarly, in Argentina, commercial honeys are ruled by Código Alimentario Argentino (CAA) and Mercado Común del Sur rules. The maximum level of microorganisms allowed by this legislation for molds and yeast with trading purposes is 100 cfu/g of honey. Likewise, the legislation does not allow the presence of *Salmonella* and *Shigella* bacteria or total coliforms in honey [16,17].

In the present study yeasts and molds were found in 11 of Thai honeys samples (55%) and the mean count of them was 1.24 log cfu/g. Eight from eleven positive honey samples (T5, T8, T9, T11, T13, T14, T18 and T19) were above the national microbiological limit (2.0 log cfu/g) [20]. The growth of the microorganisms was probably caused by elevated values of water activity of in range 0.569–0.647 and water contents of five of these eight samples (T5, T9, T11, T13 and T14) contained an above-the-limit twenty percent quantity of water (21.65–25.07%). Gomes et al., 2010 reported yeast and mold count on a similar level with Thai honey with average count of 1.53 log cfu/g [10]. Iurlina et al., 2005 showed count yeast and molds equal to or lower than 2.67 log cfu/g (57% of honey samples) [33]. Similar level of mold 2.0–2.2 log cfu/g (17% of samples) and yeast 2.07–2.99 log cfu/g examined separately showed in Mexico honey samples [16]. In the present study yeasts and mold were found in 45% and the mean count of them was 0.26 log cfu/g in the Polish honey samples. Only three samples contained some small number of yeasts and molds (0.1–0.56 log cfu/g) and above-the-limit of water content 20.5–22.0%. 

The mean values obtained for the total number of mesophilic aerobic bacteria in the tested honey samples were 0.98 and 1.13 log cfu/g for Polish and Thai honeys, respectively. Very similar results found Gomes et al., 2010, who have found that all tested honey samples were contaminated <1.0 log cfu/g of TVC except one sample which was contaminated on the level 1.30 log cfu/g [10]. Iurlina et al., 2005 reported higher level of contamination (average 2.38 log cfu/g), but still in range of no mandatory Mexico NMX-036-Normex-2006 standard [16,33]. According to Mexico standard (no more than 1000 cfu/g of nonpathogenic bacteria and up to 100 cfu/g of yeast and molds) 40.5% of honey samples from Mexico did not meet the specification in the case of aerobic mesophilic bacteria [16].

We have found that the number of LAB were higher in the Polish samples in comparison to the Thai honey. Lactic acid bacteria are recognized as safe and play important role in the preservation of the product since many of them have the ability to produce antimicrobial agents such as organic acids and bacteriocins that can inhibit or destroy pathogenic bacteria although this characteristic may depending on the type of LAB. Very few (2%) of Mexico honey samples contain LAB and only 15.79% of the samples contained more than 2 log cfu/g [16]. Earlier reports also detected the presence of some vegetative, non-spore forming lactic acid bacteria in raw honey [40,41,42,43]. *Lactobacillus, Lactococcus* and *Leuconostoc* genera were isolated from flowers, plant surfaces and plant associated products suggesting that LAB which present in honey may come from plant sources and the bees [44].

However, in the case of mesophilic spores of the *Bacillus* spp. bacterium, in the present study the mean value obtained in the case of 18 (90%) Polish honeys samples was 0.82 log cfu/g, for 16 (80%) of the 20 Thai honeys samples the mean value was 0.98 log cfu/g (Figure 2).

Moreover, other authors indicated that some opportunistic spore-forming bacteria, molds and yeasts are typically found in honey, often at low numbers, while spores can persist indefinitely [43]. Spores from the *Bacillus* genus are regularly found in honey. Iurlina et al., 2005 and Iurlina, et al., 2006 showed 23–27% *Bacillus* spp. positive honey samples which were identified as *B. cerus, B. pumilus* and *B. laterosporus* [33,45]. Some authors reported that the potential toxigenic effects of *Bacillus* were achieved with 10^4^ spores per g of honey [7]. Consumption of foods that contain more than 10^4^ spores or vegetative cells *B. cereus* per gram may results in food poisoning. *B cereus* can produce several toxins, most important being the emetic toxin and the HBL enterotoxin complex. Investigation of honey samples from Argentina, conducted various methods, found *B. cereus* in 27% samples and other species of *Bacillus* 14% of honey samples [46]. In another investigation the most frequently isolated from honey samples was *Bacillus amylioliquefacien* [20].

The microbiota of ripening honey can also pose a threat to human health, due to the viable from of pathogens as: *Klebsiella* spp., *Salmonella* spp. and *Shigella* spp. [47]. In the present study, the *Salmonella* spp. was not detected in any of the tested honey samples, which is in accordance with Regulation (EC) No 2073/2005 [48]. The same results were obtained by other authors when examining honey samples [10,16,33] and the samples coming from honey drums [17]. However, up to date no vegetative forms of disease-causing bacterial species have been found in honey. Bacteria do not replicate in honey and as such high numbers of vegetative bacteria could indicate recent contamination from a secondary source [15].

## 4. Materials and Methods

### 4.1. Research Material

The research material consisted of ripe honey samples purchased directly from beekeepers in apiaries in order to avoidance of manipulative contamination research material. The time from acquisition from the apiary to analysis did not exceed 3 months. Each sample was stored at room temperature in darkness for future study. Twenty Polish samples were representing a climate transitional between the continental moderate and oceanic moderate one (P1–P20). Honeys purchased in Thailand represented the monsoon tropical climate zone (T1–T20). In Table 1 characteristic of honey samples was shown, according to producer declaration.

### 4.2. Methods of Analysis

#### 4.2.1. Pollen Analysis

Conformity of the honey samples was determined by palynologic microscopic observations consistent with the method recommended by the International Commission for Bee Botany and by the International Honey Commission Variety and conformity with the Polish standard [28,29].

#### 4.2.2. Physicochemical Analysis

The pH value of honey was determined using Lab 860 pH meter (SI Analytics GmbH, Schott Instruments, Germany). Samples of honey were prepared by dissolving 10 g of honey in 75 mL of distilled water carbon dioxide free [28].

The water activity was measured at 25 ± 0.2 °C using Aqualab TE series 4 analyzer (Decagon Devices, Pullman, Washington, DC, USA), in a temperature stable sampling environment, calibrated with saturated salt solutions in the a_w_ range of 0.40–0.70. This device operates based on electronic dew-point measurements. AquaLab analyzer continues the analyses of water activity until the difference of three consecutive measurements is less than 0.0005 a_w_ [34].

Water content in undiluted honeys was established using PAL-22S refractometer (ConbestCo, Kraków, Poland)—three/four measurements have been performed.

#### 4.2.3. Microbial Counts Analysis

For each sample a mass of 10 g of honey was weighed aseptically and homogenized for 1 min with 90 mL of Buffered Peptone Water (BPW) in Stomacher 400 instrument (IUL Instruments, Königswinter, Germany). To perform 10-fold dilutions BPW has been used. The microbiological quality of honey samples was tested using the reference plate method, performing analysis on two parallel plates. The number of microorganisms was expressed as colony forming units per gram of honey (cfu/g).

Determination of TVC was performed using the ISO standard [49] with Plate Count Agar (PCA Biokar Diagnostics, Austria). The plates were incubated at 30 °C for 48–72 h days. The counts of yeast and mold were determined on Rose Bengal Chloramphenicol Agar (RBC Agar, Becton Dickinson and Co., Franklin Lakes, NJ, USA), after incubation at 25 °C for 5 days [50]. The number of mesophilic cells of LAB was determined with a pour plate method using MRS agar (de Man, Rogosa and Sharpe, Biokar Diagnostic, Wien, Austria). Plates were incubated anaerobically at the temperature 30 °C for up to 72 h [51]. The presence of mesophilic *Bacillus* spp. bacteria, determined using Mannitol Egg Yolk Agar supplemented with Polymyxin—Mossel (MYP) Agar (Bio-Rad, Watford, UK), after incubation at 37 °C for 24–48 h [52].

#### 4.2.4. Bacterial Detection Analysis

Detection of *Salmonella* species in 25 g was performed instrumentally using BacTrac 4300 screening impedance method (SyLabGeräte GmbH, Purkersdorf, Austria). Analysis has been performed using BiMedia 201C Salmonella culture medium (modif. Rappaport-Vassiliadis media), measurement cells were incubated at 40 °C for 24 h, threshold 10% for the E-value (Electrode-value, impedance around electrode).

#### 4.2.5. Data Analysis

The cluster analysis method and the Statistica 13.3 PL software were used to classify the results of microbiological analyses of honey [53]. The distance between clusters was measured by Euclidean distance function, while the Ward method was used to bind the clusters. The Ward method uses the assumptions of variance analysis and aims to minimize the sum of deviations within clusters. As a result of joining cluster pairs, the pair that gives the cluster with the minimum differentiation is chosen. Error sum of squares (ESS) is a measure of the difference to the mean value. The test t-student was used to assess differences between the samples (α = 0.05) and the linear coefficient of Pearson to assess the correlation between the variables.

## 5. Conclusions

The study has revealed that geographical origin of honey may significantly affect the quality and safety of honey especially from different climatic zones. Our study indicated that the Polish and the Thai honey samples, although considered as multiflower, were differ in case of count yeast and mold, which was affected by water content. It was considered that water content can be the most informative and handy marker of the microbiological quality of honeys. The classification of honeys samples found that the total number of viable microorganisms could be used as a measure alternative to the count of *Bacilus* spp., which is important hint for the legislator. Moreover, 70% of the tropical honey samples had different microbiological quality than honey from temperate climate zone. Analysis of lactic acid bacteria showed temperate climate zone honeys as a source of beneficial bacteria in the diet. 

Due to the lack of EU and World regulations on the presence of microorganisms in honey, the attention should be given to the origin of the honey. The microbiological criteria used in the legislations of different countries are the result of good production and hygiene practices applied by honey producers and constitute an indispensable reference point in assessing the quality of honey and show that microbiological control of honey is needed in order to ensure the safety of consumers.

## Figures and Tables

**Table 1:** Analyzed honey samples according to beekeepers’ declarations.

Sample No.	Producer Declaration	Sample No.	Producer Declaration
P1	goldenrod nectar honey	T1	coffee nectar honey
P2	raspberry nectar honey	T2	coffee nectar honey
P3	rapeseed nectar honey	T3	coffee nectar honey
P4	rapeseed nectar honey	T4	coffee nectar honey
P5	linden nectar honey	T5	coffee nectar honey
P6	linden nectar honey	T6	wild forest nectar honey
P7	multiflorous nectar honey	T7	wild forest nectar honey
P8	multiflorous nectar honey	T8	wild forest nectar honey
P9	multiflorous nectar honey	T9	wild forest nectar honey
P10	buckwheat nectar honey	T10	longan nectar honey
P11	multiflorous nectar honey	T11	longan nectar honey
P12	dandelion nectar honey	T12	longan nectar honey
P13	forest nectar honey	T13	longan nectar honey
P14	multiflorous nectar honey	T14	longan nectar honey
P15	raspberry nectar honey	T15	longan nectar honey
P16	buckwheat nectar honey	T16	longan nectar honey
P17	multiflorous nectar honey	T17	longan nectar honey
P18	acacia nectar honey	T18	lychee nectar honey
P19	acacia nectar honey	T19	lychee nectar honey
P20	forest nectar honey	T20	lychee nectar honey

**Table 2:** Physicochemical properties of Polish and Thai honey samples.

Polish and Thai Honey Sample			
No. of Sample	pH ± SD	Water Content [%] ± SD	Water Activity ± SD
P1	4.07 ± 0.02	20.90 ± 0.04	0.566 ± 0.00
P2	3.47 ± 0.02	17.20 ± 0.05	0.544 ± 0.00
P3	3.38 ± 0.04	22.00 ± 0.01	0.549 ± 0.00
P4	3.71 ± 0.05	16.70 ± 0.05	0.503 ± 0.00
P5	4.12 ± 0.03	19.70 ± 0.00	0.521 ± 0.00
P6	4.15 ± 0.11	16.70 ± 0.02	0.533 ± 0.01
P7	4.12 ± 0.01	18.80 ± 0.02	0.526 ± 0.00
P8	4.43 ± 0.03	20.50 ± 0.00	0.543 ± 0.00
P9	3.92 ± 0.09	18.30 ± 0.10	0.547 ± 0.02
P10	3.86 ± 0.05	19.10 ± 0.06	0.559 ± 0.01
P11	3.43 ± 0.06	19.20 ± 0.08	0.538 ± 0.01
P12	4.25 ± 0.10	16.00 ± 0.10	0.501 ± 0.00
P13	4.22 ± 0.12	16.90 ± 0.09	0.525 ± 0.00
P14	3.53 ± 0.09	15.10 ± 0.10	0.516 ± 0.00
P15	3.78 ± 0.05	19.60 ± 0.00	0.532 ± 0.01
P16	3.54 ± 0.07	20.70 ± 0.09	0.578 ± 0.00
P17	3.62 ± 0.11	19.00 ± 0.05	0.537 ± 0.00
P18	3.66 ± 0.10	17.60 ± 0.05	0.513 ± 0.00
P19	3.72 ± 0.01	17.40 ± 0.80	0.558 ± 0.00
P20	3.52 ± 0.08	16.60 ± 0.20	0.505 ± 0.00
Average value	3.75 ± 0.05	18.55 ± 0.05	0.535 ± 0.00
Median value	3.82 ± 0.06	18.40 ± 0.09	0.534 ± 0.00
T1	4.67 ± 0.01	21.80 ± 0.07	0.594 ± 0.00
T2	3.44 ± 0.03	21.70 ± 0.19	0.643 ± 0.00
T3	4.18 ± 0.03	17.31 ± 0.11	0.572 ± 0.01
T4	4.53 ± 0.00	21.33 ± 0.09	0.621 ± 0.00
T5	4.90 ± 0.09	21.65 ± 0.03	0.629 ± 0.02
T6	4.16 ± 0.01	17.86 ± 0.16	0.575 ± 0.00
T7	4.53 ± 0.00	20.87 ± 0.12	0.633 ± 0.00
T8	3.50 ± 0.04	17.14 ± 0.10	0.567 ± 0.01
T9	3.67 ± 0.03	25.07 ± 0.16	0.656 ± 0.01
T10	3.73 ± 0.11	24.21 ± 0.18	0.673 ± 0.00
T11	3.87 ± 0.06	21.70 ± 0.22	0.608 ± 0.00
T12	3.82 ± 0.08	26.20 ± 0.31	0.657 ± 0.01
T13	3.72 ± 0.10	23.31 ± 0.24	0.647 ± 0.02
T14	3.83 ± 0.14	22.20 ± 0.15	0.596 ± 0.00
T15	3.66 ± 0.20	20.50 ± 0.11	0.592 ± 0.00
T16	3.69 ± 0.08	17.40 ± 0.09	0.582 ± 0.00
T17	4.62 ± 0.03	16.00 ± 0.09	0.577 ± 0.02
T18	4.17 ± 0.10	17.60 ± 0.10	0.553 ± 0.00
T19	3.90 ± 0.12	19.50 ± 0.04	0.602 ± 0.00
T20	3.64 ± 0.01	26.41 ± 0.20	0.612 ± 0.01
Average value	3.85 ± 0.05	21.49 ± 0.11	0.605 ± 0.00
Median value	4.01 ± 0.06	20.98 ± 0.13	0.609 ± 0.00

P—Polish honey samples; T—Thai honey samples. SD-standard deviation.

## Data Availability

All data and figures generated or used during the study appear in the submitted article.
